# Clinical characteristics and prognosis of non-typhoidal *Salmonella* bacteremia in children vs. adults: a retrospective study

**DOI:** 10.3389/fmed.2025.1597371

**Published:** 2025-06-26

**Authors:** Jianwei Su, Wanping Zhong, Baofang Liang, Yanhong Wang

**Affiliations:** ^1^Department of Clinical Pharmacy, Dongguan Tungwah Hospital, Dongguan, China; ^2^Department of Healthcare-associated Infection Management, The Tenth Affiliated Hospital of Southern Medical University (Dongguan People's Hospital), Dongguan, China

**Keywords:** non-typhoidal *Salmonella*, bacteremia, clinical characteristics, hematological parameters, children

## Abstract

**Background:**

Several studies have reported the clinical characteristics of non-typhoidal Salmonella (NTS) bacteremia in children and adults. However, there is a lack of data that directly compares the clinical characteristics and prognosis in children vs. adults.

**Methods:**

A retrospective study was conducted on bloodstream infections caused by NTS in hospitalized patients from 1 January 2018 to 31 December 2024. The clinical and relevant laboratory data of patients between adult and pediatric groups were compared, and the risk factors predicting the duration of antibiotic treatment were analyzed by multivariate logistic regression.

**Results:**

In total, 52 patients with NTS bloodstream infection met the eligibility criteria, with 28 (53.8%) being children vs. 24 (46.2%) adults. Respiratory infections are the most concomitant diseases (children 78.6% vs. adults 50.0%, *p* = 0.031). More than half of pediatric patients isolated NTS from their stool (children 57.1% vs. adults 20.8%, *p* = 0.008). The duration of antibiotic treatment in children is significantly lower than that in adults {8 children (6.5, 10.5) vs. 15 adults (7.25, 21.25), *p* = 0.002}; however there is no significant difference in mortality rates. Multivariate logistic regression analysis showed that children (OR = 0.209, 95% CI: 0.058 ~ 0.751, *p* = 0.016) had a shorter course of antibiotic treatment.

**Conclusion:**

A shorter course of antibiotic treatment was observed in pediatric patients; however, due to its biases and limitations, further prospective randomized controlled trials are needed to generalize our findings.

## Introduction

Non-typhoidal *Salmonella* (NTS) is an important pathogen of gastrointestinal disease globally. Its clinical manifestations are complex and primarily cause infectious diarrhea (1). Although the symptoms of intestinal infection caused by NTS infection are typically self-limiting and can recover without treatment, bacteremia can occur in approximately 5 to 9% of patients, such as infants, the elderly, and those with compromised immune systems (2–4).

NTS can be transmitted to humans through various pathways, including the consumption of undercooked meat, products contaminated with animal excrement, contact with animals or their environment, and contaminated water (5). NTS gastroenteritis is typically believed to be obtained from animal hosts; however, the relative roles of animal hosts and human-to-human transmission of iNTS disease-causing strains remain unclear (6).

Most pediatric patients recover within 1 week; however, adult patients may exhibit differences due to varying underlying diseases (7). Some studies have reported on the clinical characteristics and changes in hematological parameters among children; however, there is a rare comparative analysis of the clinical characteristics of bacteremia between children and adults (8, 9).

In this study, we included patients diagnosed with NTS bloodstream infection who met the established inclusion and exclusion criteria. We aim to provide supportive evidence for the clinical management of NTS bloodstream infection by comparing the clinical characteristics at baseline and antibiotic use among different age groups.

## Methods

### Study design and population

This was a single-center, retrospective study at Dongguan Tungwah Hospital that included inpatients with NTS bloodstream infection from 1 January 2018 to 31 December 2024. The diagnosis of bloodstream infection must meet at least one of the following criteria: (1) NTS was cultured from one or more blood cultures, and the organism cultured from blood is not related to an infection at another site. (2) The patient has clinical evidence of infection, including fever (> 38°C), chills, or hypotension (10). All data were collected from the electronic medical records. Patients with hematological malignancies who had abnormal hematological parameters due to treatment or disease were excluded.

### Microbiological and hematological tests

All blood samples were sent to the microbiology laboratory of our hospital for culture, strain identification and *in vitro* drug sensitivity test. Based on the recommendation of the Clinical and Laboratory Standards Institute (CLSI), ciprofloxacin, trimethoprim/sulfamethoxazole, ceftriaxone, and azithromycin were used for the *in vitro* sensitivity test (11). Hematological parameters including white blood cell (WBC) count, percentage of neutrophils (NEU%), percentage of lymphocytes (LYM%), and percentage of eosinophils (EOS%) were recorded in the laboratory database.

### Research outcomes

The primary outcome was the antibiotic treatment days in children and adults, and the sensitivity of non-typhoidal Salmonella (NTS) was further explored.

### Definition of related variables

Community-acquired bacteremia was defined as bacteremia that develops in a patient prior to admission, or a positive blood culture obtained within 48 h of hospital admission. The pediatric population was defined as patients aged 16 years or less. The duration of antimicrobial treatment was defined as the total length of time that NTS-susceptible antimicrobial agents were administered in the treatment of NTS bloodstream infection, including the duration of empirical antimicrobial treatment prior to the reporting of susceptibility culture results. The time for rechecking hematological indicators is defined as more than 3 days of standardized treatment with sensitive antibiotics but less than 5 days. The severity of bacteremia was assessed using the Pitt bacteremia score (12). Patient comorbidities were assessed using the Charlson comorbidity index (CCI) (13).

### Statistical analysis

SPSS 22 software was used for statistical analysis of the data, and the counting data was expressed in percentage through the chi-square test. Measurement data conforming to normal distribution were expressed as mean ± standard deviation and compared by an independent sample *t-test*. The comparison of abnormal distribution data adopted the Mann–Whitney U-test and is expressed as M(P25, P75). A *p*-value of <0.05 indicates a statistically significant difference.

## Results

### Patients’ clinical characteristics and clinical outcomes

A total of 52 patients with NTS bloodstream infection were enrolled from 1 January 2018 to 31 December 2024, and the flow chart illustrating the study inclusion process is shown in Supplementary Figure S1. There were 28 patients (53.8%) in the children group and 24 patients (46.2%) in the adult group. The majority of patients were predominantly male (33/52, 63.5%), and the median age in the children group was 1 year old or less, compared to the adult group of 58.5 years. Fever (children: 100.0%, adults: 87.5%) and abdominal discomfort (children: 75.0%, adults: 58.3%) are the main clinical symptoms, and community-acquired infections account for a high proportion of NTS bloodstream infections. Respiratory diseases are the most common comorbidities account in children (children 78.6% vs. adults 50.0%, *p* = 0.031), followed by acute enteritis (children 53.6% vs. adults 45.8%, *p* = 0.578); however, cerebrovascular disease and malignancy were significantly more prevalent in adults (*p*<0.05). Compared with the adult group, the children group had a significantly higher positive rate from stool (children: 57.1% vs. adults: 20.8%, *p* = 0.008) and a lower duration of antibiotic therapy (median 8 days; IQR, 6.5–10.5 vs. median 15 days; IQR, 7.25–21.25, *p* = 0.002), but the mortality rate was not revealed significantly different. (shown in Table 1) There were significant differences in hematological parameters, including NEU%, LYM%, and EOS% between the two groups. In addition, the comparison of hematological parameters before and after treatment showed that the percentage of eosinophils increased significantly after treatment (Supplementary Table S1; Supplementary Figures S2, S3).

**Table 1:** Clinical features of patients with NTS bloodstream infection.

Characteristic	NTS-children (*n* = 28)	NTS-adults (*n* = 24)	*Z/χ^2^*	*p*-value
Age, mean years (P25, P75)	1 (1, 1)	58.50 (46.75, 66.50)	−6.496	**0.000**
3–12 months, *n* (%)	24 (85.7)			
<1–5 years, *n* (%)	4 (14.3)			
Male, *n* (%)	17 (60.7)	16 (66.7)	0.197	0.657
Community-acquired, *n* (%)	27 (96.4)	21 (87.5)	0.466	0.495
Underlying diseases, *n* (%)				
Respiratory disease	22 (78.6)	12 (50.0)	4.661	**0.031**
Acute enteritis	15 (53.6)	11 (45.8)	0.310	0.578
Malignancy	0 (0)	5 (20.8)	4.279	**0.039**
Cardiovascular disease	0 (0)	6 (25.0)	5.653	**0.017**
Vascular ulcer	0 (0)	4 (16.7)	2.981	0.084
Possible sources of bacteremia, *n* (%)				
Stool	16 (57.1)	5 (20.8)	7.077	**0.008**
Urine	0 (0)	1 (4.2)	0.006	0.938
Pus	0 (0)	3 (12.5)	1.771	0.183
Symptoms and signs, *n* (%)				
Fever (T ≥ 38°C)	28 (100)	21 (87.5)	1.771	0.183
Nausea and vomiting	7 (25.0)	7 (29.2)	0.114	0.736
Diarrhea and abdominal pain	21 (75.0)	14 (58.3)	1.631	0.202
Hematological parameters, *n* (%)				
WBC (≥12 × 10^9^/L)	11 (39.3)	11 (45.8)	0.227	0.634
NEU% (≤75%)	27 (96.4)	3 (12.5)	37.295	**0.000**
NEU% (≤40%)	15 (53.6)	1 (4.2)	14.808	**0.000**
LYM% (≤50%)	14 (50.0)	23 (95.8)	13.226	**0.000**
LYM% (≤20%)	3 (10.7)	22 (91.7)	33.926	**0.000**
EOS% (≤0.5%)	14 (50.0)	18 (75.0)	3.413	0.065
Antibiotic course day (P25, P75)	8 (6.5, 10.5)	15 (7.25, 21.25)	−3.093	**0.002**
30-day all-cause mortality	0 (0)	2 (8.3)	0.696	0.404

WBC, white blood cell count; NEU%, neutrophil ratio; LYM%, lymphocyte ratio; EOS%, eosinophil ratio.

Data are presented as No. (%) unless otherwise indicated. *p*-values in bold are statistically significant.

### Patients’clinical characteristics and antibiotic course day≤10 days

In the univariate analysis, although cardiovascular disease and malignant tumors did not show significant differences in shorter antibiotic treatment courses (antibiotic course day≤10 days), the CCI score suggests that higher scores have a significant impact on longer antibiotic use courses (antibiotic course>10 days) (OR = 0.792, 95% CI:0.638 ~ 0.985, *p* = 0.036). (Table 2) In the multivariate analysis for the factors significantly associated with antibiotic course day≤10 days, children indicated significant correlations with shorter antibiotic treatment courses (OR = 0.209, 95% CI: 0.058 ~ 0.751, *p* = 0.016). CCI did not show a significant difference in the impact on the short-term course of antibiotic use (OR = 0.864, 95% CI: 0.685 ~ 1.091, *p* = 0.220). (Table 3).

**Table 2:** Univariate analysis of the factors associated with the antibiotic course day.

Variables	Antibiotic course day≤10 days, *n* = 31 (%)	Antibiotic course day>10 days, *n* = 21 (%)	OR	95% CI	*p-*value
Children	22 (70.9)	6 (28.5)	0.164	0.048–0.556	**0.004**
Underlying diseases					
Respiratory disease	21 (67.7)	13 (61.9)	1.292	0.406–4.117	0.664
Acute enteritis	16 (51.6)	10 (47.6)	1.173	0.387–3.556	0.778
Malignancy	3 (9.6)	2 (9.5)	1.018	0.155–6.682	0.985
Cardiovascular disease	2 (6.4)	4 (19.0)	0.293	0.048–1.773	0.181
Possible sources of bacteremia					
Stool	13 (41.9)	8 (38.0)	1.174	0.378–3.645	0.782
Urine	1 (3.2)	0 (0)	-	-	1
Pus	0 (0)	3 (14.2)	-	-	0.999
Pitt	1.3 ± 0.5	1.3 ± 0.8	0.923	0.389–2.190	0.855
CCI	1.3 ± 2.6	3.0 ± 2.7	0.792	0.638–0.985	**0.036**

*p*-values in bold are statistically significant.

**Table 3:** Multivariate analyses of the factors associated with antibiotic course day.

Variables	Antibiotic course day≤10 days, *n* = 31 (%)	Antibiotic course day>10 days, *n* = 21 (%)	OR	95% CI	*p*-value
Children	22 (70.9)	6 (28.5)	0.209	0.058–0.751	**0.016**
CCI	1.3 ± 2.6	3.0 ± 2.7	0.864	0.685–1.091	0.220

*p*-values in bold are statistically significant.

### Antibiotic sensitivity of NTS between children and adults

There was no significant difference in the sensitivity of NTS between children and adults for first-line therapeutic drugs (Table 4). All the first-line therapeutic drug sensitivity of the adult group was higher than that of the children group, and the cases of multi-drug resistance in the children group were significantly higher than the adult group (children 42.9% vs. adults 16.7%, *p* = 0.041).

**Table 4:** Sensitivity rates of NTS to antimicrobial agents (*n*, %).

Antimicrobial agent	Total (*n* = 52)	Children (*n* = 28)	Adults (*n* = 24)	*χ^2^*	*p*-value
Ciprofloxacin	24 (46.2)	11 (39.3)	13 (54.2)	1.152	0.283
SMZ-TMP	37 (71.2)	17 (60.7)	20 (83.3)	3.221	0.073
Ceftriaxone	43 (82.7)	22 (78.6)	21 (87.5)	0.231	0.631
Ampicillin	26 (50.0)	13 (46.4)	13 (54.2)	0.310	0.578
Chloromycetin	35 (67.3)	16 (57.1)	19 (79.2)	2.849	0.091
Azithromycin	37 (71.2)	18 (64.3)	19 (79.2)	1.394	0.238
MDR	16 (30.8)	12 (42.9)	4 (16.7)	4.161	**0.041**

SMZ-TMP, sulfamethoxazole-trimethoprim; MDR, multidrug resistance.

Data are presented as No. (%) unless otherwise indicated. *p*-values in bold are statistically significant.

## Discussion

In this retrospective observational study, the antibiotic treatment course in the children group is significantly shorter than the adult group (children median, 8 days vs. adult median, 15 days), and CCI has a certain impact on the course of antibiotic use.

Currently, there is no universally agreed-upon optimal duration for the antibiotic treatment of NTS bacteremia. Most expert consensus suggests administering appropriate antibiotics for a period of 7 to 14 days, with an extension to 14 days recommended for patients with immunodeficiency (14–16). Some studies conducted on pediatric patients (aged over 1 year, with no focal extra-intestinal infections) have indicated that short-course therapy (lasting less than 10 days) is non-inferior to prolonged treatment in terms of clinical cure rate, with no metastatic complications or recurrent disease observed up to 12 months post-treatment (17, 18). Dhanoa et al. (4) included 55 patients with NTS bacteremia and found no significant difference in the duration of hospitalization between patients with and without severe immunosuppression; the average duration of hospital stay was 9 days. Megged et al. (19) conducted a retrospective analysis of 137 patients with NTS bacteremia and compared the clinical characteristics of adults and children. They found that children had higher rates of prior discharge from the emergency department, a higher rate of gastrointestinal symptoms, and a better prognosis.

Due to the significant physiological differences between pediatric and adult populations, there are notable variations in hematological parameters, particularly for children under 1 year of age (20–22). Previous studies indicated that more than half of NTS-infected patients, whether children or adults, have normal or low white blood cell levels (4, 23), which is consistent with our research results. The decrease in white blood cells and eosinophils in hematological parameters is considered to be related to the immune response characteristics between *Salmonella* and the host. During the acute phase of *Salmonella* infection, the bacteria can evade the innate immune response of the intestine without inducing significant neutrophil aggregation and chemotaxis (24, 25). Before treatment, over half of the eosinophils in both the adult and children’s groups were below the normal value of EOS% (≤0.5%), but following treatment, the eosinophil levels significantly increased with notable differences. (Supplementary Table S1).

In drug-sensitive testing, the sensitivity of traditional agents to NTS among children is generally lower than adults, but there is no significant difference. Ceftriaxone, azithromycin, and SMZ-TMP maintained high sensitivity in both the pediatric and adult groups, consistent with the first-line treatment plan for NTS recommended by experts (26). It is worth noting that the number of MDR cases in the children’s group is significantly higher than in adults, with a significant statistical difference. In this situation, these first-line antimicrobials have become less effective for some patients, particularly children who are not suitable for quinolones. Other alternative drugs, such as cefoperazone/sulbactam, piperacillin/tazobactam, and imipenem, have shown very high sensitivity to NTS, providing a meaningful choice for the treatment of NTS with MDR (27).

Given the special nature of retrospective analysis, a major limitation of the present study was that it was retrospective with a small sample size and the lack of unified reference standards for hematological parameters in children under 1 year old. Moreover, adult patients were more likely to be accompanied by chronic diseases such as respiratory, cardiovascular diseases, or malignancies, which may cause bias in the statistical results of the course of antibiotic use.

## Conclusion

Our study demonstrates that pediatric patients may consider receiving a shorter course of antibiotics compared to adult patients, and eosinophils can serve as important hematological indicators for predicting NTS infection across all age groups.

## Data Availability

The original contributions presented in the study are included in the article/Supplementary material, further inquiries can be directed to the corresponding author.
