# Supplementary material for: Clinical characteristics and prognosis of non-typhoidal Salmonella bacteremia in children vs. adults: a retrospective study
Source: Front Med (Lausanne). 2025 Jun 26;12:1597371. doi: 10.3389/fmed.2025.1597371 (PMC12240969; doi:10.3389/fmed.2025.1597371)
Supplement: Supplementary file 1 [file Data_Sheet_1.docx]

**Supplemental content for**

**Clinical characteristics and prognosis of non-typhoidal *Salmonella* bacteremia in children versus adults:** **A retrospective study**

**Figure S1** study flow chart

**Table S1** Comparison of hematological parameters before and after treatment

**Figure S2** Changes in eosinophil percentage before and after treatment

**Figure S3** Numbers of eosinophil percentage＜0.5 before and after treatment


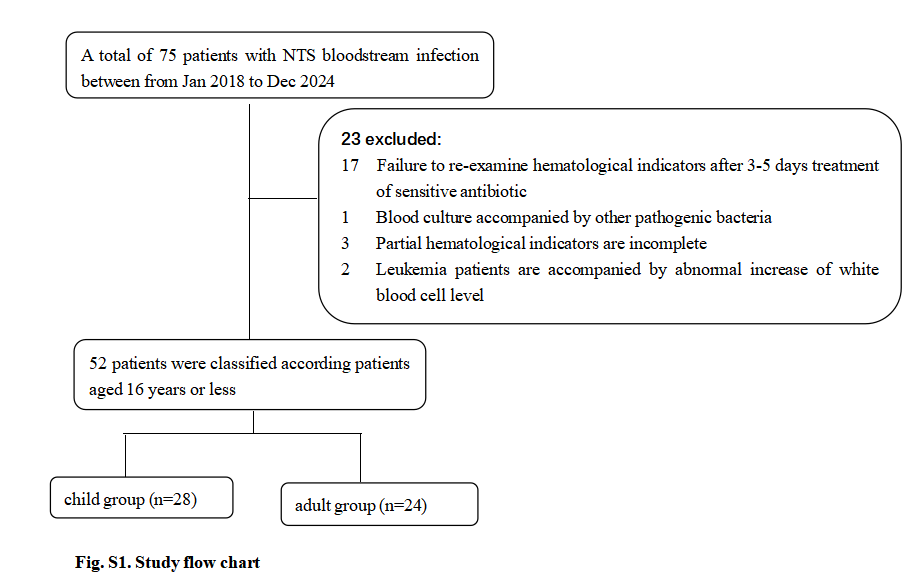


TABLE S1 Comparison of hematological parameters before and after treatment

| Haematological changes | Child（n=28） | | *Z/χ^2^* | *P*-value | Adult（n=24） | | *Z/χ^2^* | *P*-value |
| --- | --- | --- | --- | --- | --- | --- | --- | --- |
|  | Before^a^ | After^b^ |  |  | Before^a^ | After^b^ |  |  |
| WBC(×10^9^/L) | 10.82(7.81,14.88) | 9.38(6.99,12.36) | -1.799 | **0.000** | 9.5(4.7,12.99) | 6.67(4.81,10.95) | -1.416 | 0.157 |
| WBC(≥12×10^9^/L) | 11(39.3) | 8(28.6) | 0.094 | 0.760 | 11(45.8) | 5(20.8) | 2.304 | **0.031** |
| NEU% | 3.95(2.38,7.51) | 2.52(1.73,4.32) | -2.476 | **0.013** | 8.68(3.28,11.16) | 4.1(2.94,6.73) | -2.703 | **0.007** |
| NEU%(≤75%) | 27(96.4) | 28(100) | - | - | 3(12.5) | 17(70.8) | -5.675 | **0.000** |
| NEU%(≤40%) | 15(53.6) | 20(71.4) | 0.434 | 0.510 | 1(4.2) | 3(12.5) | -1.446 | 0.162 |
| LYM% | 46.48±18.36 | 58.13±15.71 | -2.950 | **0.006** | 6.1(3.55,13.25) | 17.4(8.65,29.4) | -4.271 | **0.000** |
| LYM%(≤50%) | 14(50.0) | 8(28.6) | 0.175 | 0.676 | 23(95.8) | 22(91.7) | 1 | 0.328 |
| LYM%(≤20%) | 3(10.7) | 1(3.6) | 1.673 | 0.196 | 22(91.7) | 12(50.0) | 4.053 | **0.000** |
| EOS% | 0.5(0.075,1.75) | 2.4(0.7,3.45) | -3.268 | **0.001** | 0.1(0,0.6) | 1.8(0.7,2.65) | -2.802 | **0.005** |
| EOS%(≤0.5%) | 14(50.0) | 5(17.9) | 3.896 | **0.048** | 18(75.0) | 6(25.0) | 3.715 | **0.001** |
| (1) Abbreviations: WBC, white blood cell count; NEU%, neutrophil ratio; LYM%, lymphocytes ratio; EOS%, eosinophils ratio.  (2) Data are presented as No. (%) unless otherwise indicated. P-values in bold are statistically significant.  (3) a. Before: indicators before receiving sensitive drug treatment; b. After: indicators after receiving standardized treatment with sensitive antibiotics for more than 3 days and less than 5 days. | | | | | | | | |


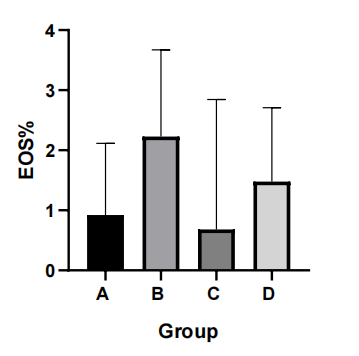


**A**: Percentage of eosinophils in children before treatment.

B:Percentage of eosinophils in children after treatment

C:Percentage of eosinophils in adult group before treatment

D:Percentage of eosinophils in adult group after treatment

**Fig. S2** Changes in eosinophil percentage before and after treatment


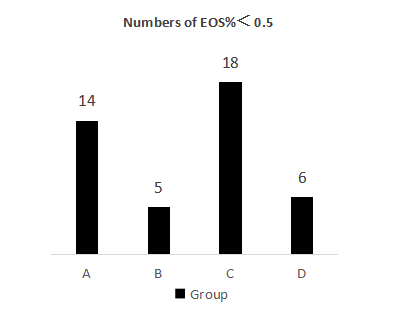


**A**: Number of children with eosinophil percentage<0.5 before treatment

B:Number of children with eosinophil percentage<0.5 after treatment

C:Number of adult with eosinophil percentage<0.5 before treatment

D:Number of adult with eosinophil percentage<0.5 after treatment

**Fig. S3** Numbers of eosinophil percentage＜0.5 before and after treatment
